# The inverse association of serum HBV DNA level with HDL and adiponectin in chronic hepatitis B infection

**DOI:** 10.1186/1743-422X-7-228

**Published:** 2010-09-14

**Authors:** Ashraf Mohamadkhani, Kourosh Sayemiri, Reza Ghanbari, Elham Elahi, Hossein Poustchi, Ghodratollah Montazeri

**Affiliations:** 1 Digestive Disease Research Centre, Shariati Hospital, Tehran University of Medical Science, Tehran, Iran; 2 Epidemiology and social medicine department, Ilam University of Medical sciences, Ilam, Iran

## Abstract

**BACKGROUND:**

The natural history of hepatitis B virus (HBV) is complex and influenced by the level of viral replication and host factors. The hepatoprotective role of high density lipoproteins (HDL) and adiponectin as host factors on HBV persistence is less well understood.

**METHODS:**

To investigate correlation between HBV DNA level with clinical parameters in patients with chronic hepatitis B, 92 male subjects with HBV infection without any risk factors for diabetes were enrolled in this study. Age and BMI of the study population were matched and HBV DNA, ALT, tumor necrosis factor alpha (TNF-α), adiponectin and lipid levels was measured.

**RESULTS:**

Serum HBV DNA correlated inversely with serum HDL level (r = -0.23; P = 0.014). The median of log copies/ml for HBV DNA (3.67) was considered as cut off point. Patients with HBV DNA level higher than cut off point had lower adiponectin (8.7 ± 5.3 vs 10.7 ± 4.9 μg/ml p = 0.05). Also, adiponectin had a negative correlation with TNF-α (r = -0.21, P = 0.04) and positive correlations with HDL (r = 0.18, P = 0.043).Multivariate regression models show that serum HDL level is an independed factor to predict serum HBV DNA.

**CONCLUSION:**

Our findings showed that higher HBV DNA levels are associated with lower HDL and adiponectin but induced TNF-alpha values.

## Introduction

The broad outcomes of hepatitis B virus (HBV) infection can be divided into acute infection and chronic hepatitis [1]. The ongoing replication of HBV in chronic hepatitis induce oxidative stress and associated with liver inflammation, which over the course of years increases risk of fibrosis, cirrhosis, and liver cancer [2,3]. Factors which determine viral replication and outcome of infection are not fully understood, although host factors are known to play a major role in this regard [4]. Among these factors, HDL, with several biological properties, including anti oxidative and anti inflammatory activities has a potential role to be a part of nonspecific immunity [5,6]. Adiponectin also attenuates inflammation, oxidative stress, and pro-inflammatory cytokine production [7].

The anti-inflammatory function of HDL involves induction transforming growth factor β which might function as an important mediator of the anti-inflammatory activity [8]. In chronic hepatitis B patients, serum HDL concentration inversely correlates with excess release of pro-inflammatory response and progressive liver disease [9,10]. Adiponectin, an adipose tissue-specific protein puts multiple beneficial effects on tissue and vascular physiology. At physiological concentrations, adiponectin suppresses TNF-α induced NF-β activation and blocks TNF-α release in endothelial cells [7]. It also ameliorates liver fibrosis via suppression of activated hepatic stellate cells function, and might slow down progression of hepatocarcinogenesis via suppression of oxidative stress [11]. In addition, adiponectin activates peroxisome proliferator-activated receptor-a (PPAR-a) to up-regulates the hepatic expression of apoproteins A-I and A-II, which in turn promotes increased hepatic HDL secretion [12,13]. Animal models revealed the hepatoprotective action of adiponectin [14]. It was shown that adiponectin exert its hepatoprotective function by increasing activation of mitochondrial fatty acids β-oxidation and thus reductions of circulation free fatty acid [13,15].

HDL and adiponectin share common metabolic pathways, however, their role in liver diseases associated with chronic hepatitis B infection is not well understood. To further investigate association of HDL and adiponectin with HBV replication rate and therefore introducing a possible strategy for treating patients with chronic hepatitis B, we examined serum HBV DNA level with regard to serum HDL concentration as well as adiponectin and proposing their beneficial function in treating chronic hepatitis B.

## Materials and methods

### Patients

Sera from 92 male adult carriers, positive for HBsAg and HBV DNA and negative for HBeAg as well as HCV, HDV, and HIV antibodies referring to a tertiary university-based referral center between September 2006 and November 2008 for regularly follow up were collected. None of the patients had evidence of cirrhosis and/or hepatocellular carcinoma. There was no clinical or para-clinical evidence of diabetes mellitus based on criteria defined by World Health Organization [16]. Both normal and elevated ALTs were included in the study. Patients had no history of treatment for HBV prior to the study and they also had no alcohol consumption. Study protocol was approved by the Ethics Committee of Tehran University of Medical Sciences (TUMS). Informed consent was obtained from all participant.

### Clinical and Laboratory Assessments

Following to an overnight fasting (12h) serum ALT, total cholesterol, triglycerides, high-density lipoprotein (HDL), fasting blood sugar (FBS), Albumin and Bilirubin were measured with commercial kits using a Hitachi 7250 special autoanalyzer (Hitachi, Tokyo, Japan). The body mass index (BMI) was calculated by the formula: weight (kg)/height (m^2^).

### Determination of Serum Adiponectin Level and TNF-α

Both serum adiponectin and TNF-α were measured using enzyme immunoassay Orgenium (Vantaa, Finland) for adiponectin and Bender Medsystems (Vienna, Austria) for TNF-α.

### Virological Assessment

HBs- and HBe- Antigens were tested using commercially available enzyme-linked immunosorbent assay kits from RADIM (Italy). HBV DNA was extracted from 200 μl of serum using QIAamp DNA Blood Mini Kit (QIAGEN USA) according to the manufacturer's instructions. HBV DNA was then quantified in the Light-Cycler (Roche) using the RealART™ HBV LC PCR (QIAGEN, Hilden, Germany) according to the manufacturer's instructions. The linear range of this assay was 10^2^-10^9 ^copies/ml.

### Statistical Analysis

Results were expressed as mean ± standard deviation. Relations between continuous variables were tested using Spearman's rank correlation coefficients, Pearson correlation coefficients and multivariate linear regression models. Adjustment on age, ALT and BMI was performed using multivariate regression models. Kolmogrov-Smirno test was used to test the normality in continues variables (Ramlu-Hansen 1983). When normal distribution assumption was not met for some variables, the square root transformation was used to change continues variables to normal distribution. Log copies/ml HBV DNA was considered as a continues variable in multivariate regression models and when it was considered as a dichotomous variable (less than median and more than median) logistic regression models was used to check association between HBV DNA and other variables. Mean differences were tested by Student's t-test. To find nonlinear association between HBV DNA and HDL with adiponectin in scatter plots, Epanechnikov kernel smooting was used. Analyses were done using STATA ver. 10. P-value less than .05 was considered significant.

## Results

### Characteristics and Clinical Parameters of study population

Demographic and biochemical characteristics of 92 consecutive middle-aged and normal weight male subjects with chronic hepatitis B are presented in Table 1. Mean of serum HBV DNA, HDL and ALT level were 3.7 ± 0.9 log copies/ml, 49 ± 11mg/dl and 48 ± 51 IU/l and for serum adiponectin and TNF-alpha were 9.7 ± 5.2 ng/ml and 10.6 ± 3.6 ng/L respectively.

**Table 1:** Clinical and biochemical characteristics of patients

***Characteristics of patients (n = 92)***	***Mean ± SD***
Age(Years)	39 ± 10
BMI (kg/m2)	25.5 ± 1.2
FBS (mg/dl)	85 ± 13
Albumin (g/dl)	4.3 ± 0.5
Bilirubin total (mg%)	0.6 ± 0.3
ALT (IU/l)	48 ± 51
Cholesterol (mg/dl)	168 ± 33
Triglyceride (mg/dl)	138 ± 42
HDL (mg/dl)	49 ± 11
Adiponectin (ng/ml)	9.7 ± 5.2
TNF-alpha (ng/L)	10.6 ± 3.6
HBV DNA (log copies/ml)	3.7 ± 0.9

### Biochemical Evaluation in Patients with Chronic Hepatitis B

Serum HBV DNA (log copies/ml) associated with ALT level (r = 0.3, P = 0.001). A negative correlation was noted between HBV DNA and serum HDL (r = -0.24, P = 0.014). The same correlation was found between adiponectin with TNF-alpha and triglyceride (r = -0.21, P = 0.04, r = -0.21, P = 0.037). In contrast, serum adiponectin showed a positive correlation with serum HDL levels (r = 0.21, P = 0.05). There was no statistically significant association between serum HDL and adiponectin with FBS, Albumin, Bilirubin, BMI, age, ALT and cholesterol.

### Association of Serum HDL and Adiponectin with HBV DNA

Analysis with median 3.67 for log copies/ml HBV DNA -set as a cut off point- showed that patients with HBV DNA higher than this level had higher levels of ALT (61 ± 65 vs 36 ± 26 p = 0.02), but lower HDL and adiponectin (47 ± 10 vs 52 ± 12 p = 0.04 and 8.7 ± 5.3 vs 10.7 ± 4.9 p = 0.05 respectively) (Table 2). When Log HBV DNA was considered as a continuous variable in a multivariate regression model we estimate equation:

**Table 2:** Association of clinical findings of chronic hepatitis B with HBV DNA level higher than median log copies/ml or lower than median (3.67 log copies/ml).

**Clinical factor**	HBV DNA < median *	HBV DNA > median *	***P value***
**Age (Years)**	41 ± 10	38 ± 11	*0.12*
**BMI (kg/m2)**	25.4 ± 1.1	25.6 ± 1.2	*0.47*
**FBS (mg/dl)**	83 ± 14	86 ± 8	*0.2*
**Albumin (g/dl)**	4.3 ± 0.5	4.2 ± 0.5	*0.7*
**Bilirubin total (mg%)**	0.5 ± 0.2	0.6 ± 0.2	*0.7*
**ALT (IU/l)**	36 ± 26	61 ± 65	*0.021*
**Cholesterol (mg/dl)**	172 ± 31	164 ± 34	*0.27*
**Triglyceride (mg/dl)**	136 ± 44	140 ± 39	*0.62*
**HDL (mg/dl)**	52 ± 12	47 ± 10	*0.04*
**Adiponectin (μg/ml)**	10.7 ± 4.9	8.7 ± 5.3	*0.057*
**TNF-a (ng/L)**	9.7 ± 2.7	11.5 ± 4.2	*0.017*

*Mean ± SD

Log HBV DNA= 4.4 -0.029 adiponectin -0.014 HDL+0.006 ALT

This regression equation shows that with considering adjustment on ALT variable, increasing HDL and adiponectin variables decrease Log HBV DNA.

TNF-α was significantly elevated in patients with HBV DNA levels more than median compared with those in HBV DNA levels less than median (11.5 ± 4.2 vs 9.7 ± 2.7 p = 0.017).

We found that 31% of patients with HBV DNA higher than median had serum HDL less than 40 mg/dl, while 15% of patients with HBV DNA lower than median had serum HDL less than 40 mg/dl. Although the Chi-squared P value was 0.06, but it was identified that the number of patients with HDL less than 40 mg/dl and HBV DNA higher than median is approximately 2 times more compared to patients who have HDL less than 40 mg/dl and HBV DNA lower than median.

In a multivariate regression model with depended variable Log HBV DNA and independed variables serum HDL, age, and BMI ,correlation between Log HBV and serum HDL was statistically significant (P = 0.036. b = -0.018). Coefficient of regression analysis showed an increased unit of serum HDL accompanied with 1.8 percent reduction in log HBV DNA. When adjustment was done on ALT in the multivariate regression model, the association between Log HBV DNA and serum HDL was significant ( P = 0.046, b = -0.016).

**Figure 1:**
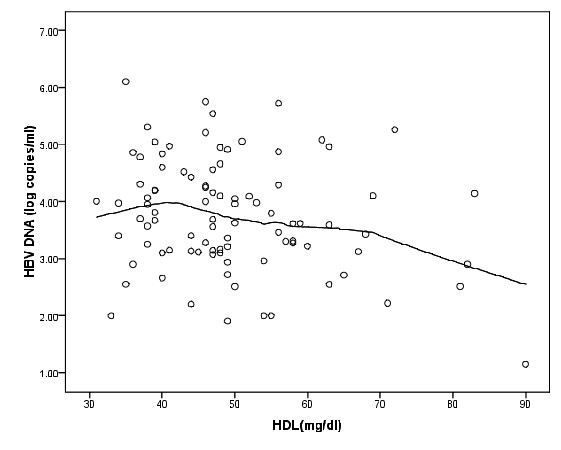
**Scatter plot of HDL (mg/dl) and HBV DNA (log copies/ml) with Epanechnikov kernel smoothing line**.

**Figure 2:**
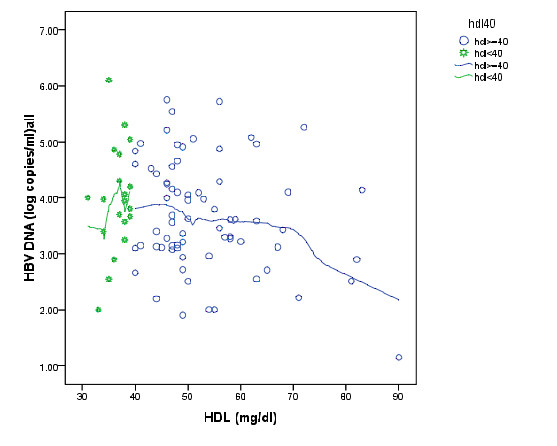
**Scatter plot of HDL (mg/dl) and HBV DNA (log copies/ml) with Epanechnikov kernel smoothing line in HDL less than 40 (star mark) and HDL more than 40 (cycle marks)**.

Epanechnikov kernel smoothing showed that, patients with serum HDL less than 40, had not a significant association with HBV DNA (r = 0.251,P = 0.76,n = 21) but in patients with serum HDL more than 40 this association was negative (r = -0.247,P = 0.038,n = 71) (Figure 1 and 2). Therefore, HBV DNA considered as a continuous variable in linear regression and a dichotomous variable in logistic regression (less than 3.67 and more than 3.67), however, the result of two methods was approximately same.

**Figure 3:**
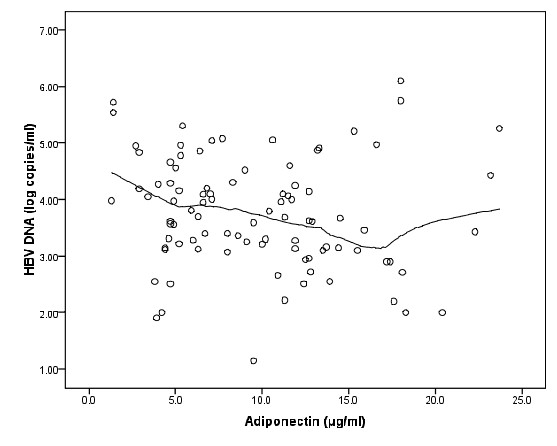
**Scatter plot of adiponectin (μg/ml) and HBV DNA (log copies/ml) with Epanechnikov kernel smoothing line**.

According to Epanechnikov kernel smoothing in figure 3 we choose 16 as a cut off point for adiponectin. Eighty patients with adiponectin less than 16, had a negative association with HBV DNA (r = -0.22, P = 0.049, n = 80). Regression equation shows that each unit increases in adiponectin accompanied with 0.05 unit decreases in log HBV DNA (t = 2.004, P = .049).

**Figure 4:**
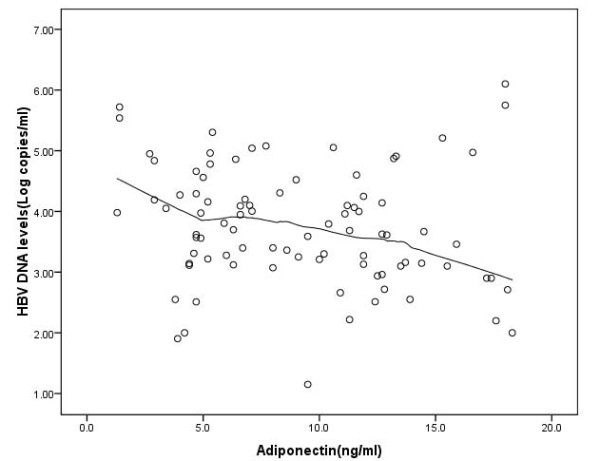
**Scatter plot of adiponectin (μg/ml) and HBV DNA (log copies/ml) with Epanechnikov kernel smoothing line when patient's adiponectin with adiponectin more than 20 were omitted**.

There were a few cases that had high levels of adiponectin, after omitting these cases there was negative correlation between Log HBV DNA and serum HDL (r = -0.27,P = 0.01 n = 88) and adiponectin (r = -0.17,P = 0.11, n = 88) figure 4 shows this relationship.

## Discussion

Development of cirrhosis and HCC has been attributed to the higher replication index of HBV [17-19]. Thus serum HBV DNA could play important role for monitoring outcome of chronic hepatitis B infection which in part seems to be affected by host factors. The key finding of our investigation was significant negative correlation between HDL and HBV DNA. Moreover we showed that there was a negative association between HBV DNA and adiponectin in a concentration less than 16 μg/ml. These associations indicating that HDL can itself reduce viral infection by potent anti-infectious activity [5]. HDL was shown to be part of nonspecific innate immunity. Changes including altered HDL content and HDL apolipoprotein composition might redirect cholesterol from the liver to immune cells during infection [5,20].

Regarding to viral infectious diseases; influenza infection accompanies with decreased levels of HDL [21]. It has also been reported that HIV-1 RNA viral load correlates with low HDL-Cholesterol levels [22]. In addition high levels of HDL-cholesterol associate with a better viral response in treated HIV patients [23]. A study carried out in Asian chronic hepatitis C patients, demonstrated that HDL had a significant effect on early viral load decline [24]. Finally most other studies have observed decreased levels of high-density lipoprotein-cholesterol in chronic hepatitis B virus infection [10,25]. HDL restores the hepatice endothelial nitric oxide synthase (eNOS) activity which is down regulated in cirrhotic patients. Hence, Thabut et al, showed limited anti-infectious activity in cirrhotic rats with decreased circulating HDL [26]. Herein, we demonstrate the protective effects of HDL to keep lower HBV DNA. It is probable that HDL is a component of the innate immune system that can limit infections [27]. The interaction of high-density lipoproteins with human neutrophils has been studied *in vitro *[28], here we propose *in vivo*, in addition to macrophages, HBV might be transferred to HDL and endocytosed within neutrophils.

As noted earlier, Adiponectin likely promotes HDL formation on multiple fronts [13]. We have now shown that adiponectin, independent from common metabolic risk factors contributes inversely with regards to HBV DNA. The positive relationship of adiponectin with the metabolic profile in adults has been less studied in chronic hepatitis B patients. Hui and co-worker reported that serum adiponectin may have a role in fibrosis progression in CHB infection [29]. In contrast another study has suggested that increased serum adiponectin in hepatic inflammatory activity which is an antagonizing TNF-α, may be a secondary to the response to hepatic injury in chronic hepatitis B [9]. Cytokines including TNF-α, are mediators of inflammation and appears to have a direct effect to inhibit apolipoproteins production by hepatocytes [30].

In summary, while the association between log HBV-DNA and serum HDL was not linear, patients with serum HDL more than 40 mg/dl showed a negative correlation with HBV DNA. In this study we adjusted the population under study for age, sex, BMI, and risk factors for diabetes which inversely correlate with serum HDL and adiponectin level [31] in order to obtain more reliable results when assessing the association of variables with the amount of HBV DNA. Thus our investigation suggests that serum HDL concentration could play inhibitory function for hepatitis B viral replication. In addition, adiponectin may also participate in the reduction of viral replication in CHB patients through activation of HDL production. However it should be noted that data regarding to association between serum adiponectin with the circulating HBV DNA level is still rather conflicting. Therefore, this association should be studied in prospective, carefully designed group controlled studies including large cohorts of patients with meticulous consideration of metabolic and other potential confounding factors.

## Competing interests

The authors declare that they have no competing interests.

## Authors' contributions

AM was responsible for doing quantitative HBV DNA, ELISA based assays and writing of this manuscript, Statistical analysis performed by KS, RG was responsible for biochemistry analysis, EE carried out for patients BMI, HP and GM visited patients as a hepatologist, coordinated for sample collection and contributed with critical reading,. The present study had financial support of Digestive Disease Research Center, Shariati hospital, Tehran University of Medical Sciences and was approved by Ethics Committee of Tehran University of Medical Sciences. contributions: All authors read and approved the final manuscript.
